# Virologic failure and HIV drug resistance on simplified, dolutegravir-based maintenance therapy: Systematic review and meta-analysis

**DOI:** 10.12688/f1000research.15995.2

**Published:** 2019-04-03

**Authors:** Gilles Wandeler, Marta Buzzi, Nanina Anderegg, Delphine Sculier, Charles Béguelin, Matthias Egger, Alexandra Calmy

**Affiliations:** 1 Department of Infectious Diseases, Bern University Hospital, Bern, 3010, Switzerland; 2 Institute of Social and Preventive Medicine, University of Bern, Bern, 3012, Switzerland; 3 Division of Infectious Diseases, Geneva University Hospital, Geneva, 1205, Switzerland

**Keywords:** Dolutegravir, simplified therapy, HIV, meta-analysis

## Abstract

**Background: **Dolutegravir-containing maintenance therapy is a promising simplification strategy for virologically suppressed HIV-infected individuals. However, most of the available data to inform this strategy come from small, uncontrolled studies. We estimated the proportion of HIV-infected patients experiencing virological failure (VF) and developing drug resistance on dolutegravir (DTG)-based maintenance therapy.

**Methods: **We searched Medline, Embase, Cochrane Central, Web of Science, and conference abstracts for studies assessing VF on DTG-based maintenance therapy. Studies including ≥5 adults with an undetectable viral load on antiretroviral therapy (ART) who switched to a DTG-based mono- or dual therapy were included. Pooled proportions of VF were estimated using random-intercept logistic meta-regression and acquired drug resistance mutations described for each strategy.

**Results**: Of 1719 studies considered, 21 met our selection criteria, including seven interventional and 14 observational studies. Eight studies including 251 patients assessed VF on DTG monotherapy and fourteen studies including 1670 participants VF on dual therapy. The participant’s median age ranged from 43 to 63 years, their median nadir CD4 count from 90 to 399 cells/µl, and 27.6% were female. The proportion of participants experiencing VF on DTG-monotherapy was 3.6% (95% confidence interval [CI] 1.9-6.7) at 24 weeks and 8.9% (95% CI 4.7-16.2) at 48 weeks. Resistance mutations developed in seven (3.6%) participants on DTG-monotherapy. Among patients on dual therapy, ten (0.7%, 95% CI 0.4-1.3) experienced VF by 48 weeks and none developed resistance to DTG. In adjusted analyses, VF at 24 weeks was less likely on dual therapy than on monotherapy (adjusted odds ratio: 0.10, 95% CI 0.03-0.30).

**Conclusions:** Whereas VF is relatively common on DTG maintenance monotherapy, DTG-based dual therapy appears to be a promising simplification strategy for individuals with a suppressed HIV viral load on triple-ART.

## Introduction

The concept of combination antiretroviral therapy (ART) for the treatment of HIV infection was established twenty years ago, when the results of the first studies evaluating protease inhibitor-based regimens were published ^1^. In recent years, several strategies of treatment optimization and simplification gained interest, with the objectives of improving quality of life, minimizing ART-related toxicity and drug-drug interactions (DDI), as well as reducing health-related costs. So far, ART de-escalation from three to one (mono-) or two drugs (dual-) therapies has mainly been evaluated in virologically suppressed patients. The first simplified maintenance strategy studied included a boosted protease inhibitor (bPI), with the hope that the high genetic barrier to resistance would help achieve durable virological suppression. In a meta-analysis including ten studies, bPI monotherapy was found to be inferior to triple ART for the maintenance of viral suppression ^2^, but non-inferior with regards to loss of future treatment options ^3^. In contrast, dual therapy with bPI and lamivudine (3TC) was found to be non-inferior to triple ART ^4– 6^ ** and is now recognized as a valid switch strategy by current HIV treatment guidelines in selected situations ^7^. However, bPI-based maintenance strategies are not widely applicable because of cost, toxicity and DDI.

Due to its interesting pharmacokinetic profile, good tolerability and high barrier to resistance, dolutegravir (DTG), a new integrase strand transfer inhibitor (InSTI), has attracted much interest for its use in simplified treatment regimens. While preliminary analyses of a Dutch DTG monotherapy simplification trial seemed encouraging at 24 weeks, rates of virological failures increased significantly by week 48, suggesting a sub-optimal potency of this regimen ^8^. On the other hand, several studies evaluating DTG-based dual therapy with either 3TC or rilpivirine (RPV), showed a high virological efficacy ^9– 12^. However, most reports were from small, observational cohort studies, with the exception of one DTG-RPV industry-sponsored randomized controlled trial (RCT) ^11^.

We performed a systematic review of the literature and a meta-analysis to provide precise estimates of the rate of virological failure (VF) and drug resistance in patients switched to a DTG-based maintenance mono- or dual therapy, and to clarify which drugs or combinations should be evaluated in further studies and implemented in clinical practice.

## Methods

The protocol for this systematic review was written and registered with the International Prospective Register of Systematic Reviews (PROSPERO registration number CRD42017070045) ^13^. The reporting of the review followed the PRISMA guidelines ^14^ ( Supplementary File 1).

### Search strategy and selection criteria

We searched Medline, EMBASE, Cochrane Central and Web of Science, as well as abstracts of major HIV conferences (CROI, AIDS, HIV Glasgow, AFRAVIH, IAS and EACS between 2013 and 2017) on 4. January 2018 for studies assessing the proportion of individuals developing VF on DTG-based maintenance therapy. In Medline we combined free text words and medical subject headings (MESH) describing the study population and the outcome ( Supplementary File 2). This search strategy was adapted for the other databases. We considered RCTs, single-arm clinical trials, cohort studies, and case-series that included at least five HIV-infected adults (≥18 years) on DTG-based simplified therapy. No language restrictions were applied. Studies had to report on virological outcomes of patients who switched to a DTG monotherapy or dual therapy after having an undetectable VL on triple ART. We excluded studies that only reported *in vitro* data and those selecting participants based on the outcome during DTG-based maintenance therapy. Two investigators (MB and GW) independently selected studies based on titles and abstracts, and, in a second step, based on the full text of potentially eligible articles. Discrepancies in study selection were resolved through discussions with a third investigator (AC).

### Data extraction

The following data were extracted independently for each study by two reviewers (GW and MB), using a standardized spreadsheet: bibliographic details, study design, inclusion and exclusion criteria, definitions of outcomes, country, number of participants and their main demographic and clinical characteristics, including duration since HIV diagnosis, ART history, immunological status (CD4 cell count at switch and nadir) and virological parameters (HIV RNA peak and at baseline, HIV-DNA at baseline and changes during the study, VF as defined by the study, and the presence at drug resistance at switch). Again, discrepancies in data extracted were resolved through discussions with a third investigator (AC).

### Assessment of risk of bias

A checklist for the assessment of risk of bias was designed to ensure data quality assessment for each study was included. The form for RCTs included information on the sequence generation, allocation concealment, blinding (participants, personnel and outcome assessor), incomplete outcome data, selective outcome reporting and other sources of bias. The methodological components of the randomized trials were assessed by two independent authors and classified as high, low or unclear risk of bias, as recommended by the Cochrane collaboration ^15^. For observational studies it was not appropriate to use the ROBINS-I tool ^16^, as we only considered data from the group of patients on simplified, maintenance therapy. Thus, we assessed the population characteristics and missing outcome data for each study.

### Data analysis

We described the study design as well as the demographic and clinical characteristics of the population from each study by type of maintenance therapy (DTG-based monotherapy or dual therapy). Pooled proportions of VF and treatment failure (VF or departure from simplified strategy due to toxicity, loss to follow-up, patient’s or physician’s decision), and 95% confidence intervals (CI) were estimated using random intercept logistic meta-regression. These analyses were performed separately at 24 weeks and 48 weeks after the switch from triple ART to maintenance therapy. For all models, statistical evidence for heterogeneity between studies was assessed using the tau-squared statistics ^17^. Of note, we separated data from Blanco *et al.* ^18^ according to the strategy used (DTG-monotherapy and DTG-based dual therapy). We evaluated the association between type of maintenance strategy and VF using random intercept logistic meta-regression (binomial-normal) models. All models were adjusted for potential confounders, including age (median or mean), sex (proportion of female participants) and study type (interventional or observational). Furthermore, the proportion of participants acquiring new drug resistance mutations was assessed for each treatment strategy and the mutations described in detail. Statistical analyses were conducted in STATA version 14.1 (StataCorp, Texas, USA) and R version 3.2.3 (R Core Team, Vienna, Austria).

### Role of the funding source

The funder of the study had no role in the design, data collection, data analysis, data interpretation of the results or writing of the report. The corresponding author had full access to all the data in the study and had final responsibility for the decision to submit for publication.

## Results

### Study and participant characteristics

**Figure 1.**
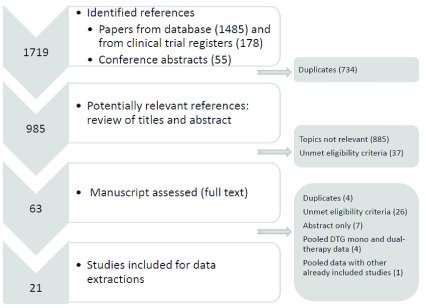
Flow chart of study selection process.

Of 1719 single studies identified, 63 remained potentially eligible after the screening of titles and abstracts. Of these, 21 studies, including four RCTs, three single-arm clinical trials and 14 observational studies met our inclusion criteria ^8, 11, 12, 18– 35^ ( Figure 1). A description of the main study characteristics by type of maintenance strategy is given in Table 1. Eight studies (two from France, two from The Netherlands, one from Germany, one from Switzerland and two from Spain) including 251 patients assessed the switch to DTG monotherapy and 14 (five from Italy, four from France, three for Spain, one from US, and one multi-country study), including 1670 participants, the switch to DTG-based dual therapy. Dual therapy consisted of DTG + 3TC (seven studies) or RPV (four studies) or atazanavir (ATV, two studies) or darunavir (DRV, one study). Overall, 14 studies allowed the inclusion of patients with previous virological failure, including five monotherapy studies ^18– 20, 22– 24^. In one study, patients with previous InSTI failure were also included ^12^. Nineteen studies assessed virological outcomes at six months of maintenance therapy, whereas ten of them additionally showed outcomes at one year ^8, 11, 12, 24, 27, 29– 33, 35^. Two studies assessed virological outcomes only at 48 weeks ^25, 27^. Median (or mean) age of participants included in the studies varied from 43 years ^11^ to 63 years ^24^ and 27.6% of them were female. 16 studies reported on the median nadir CD4 cell count, which ranged from 90 cells/µl ^12^ to 399 cells/µl ^29^.

**Table 1.** Study characteristics, by treatment group.

Study	Country	Patients included (N)	Median/ mean age (years)	Female (%)	VF definition	Study type	Eligibility criteria					
							Previous VF allowed	Previous resistances allowed	Months of stable ART	Months with HIV VL<50	CD4 nadir (cells/ µl)	Other
**DTG-Mono**												
Katlama *et al*. *JAC 2016*	F	28	48	46.4	2× ≥ 50 cp/ml or 1× > 200 cp/ml	O	Yes	-	-	≥ 12	-	-
Wijting *et al*. *Lancet HIV*	NL	96	45.5	8.5	2× ≥ 200 cp/ml	I	No	No	-	≥ 6	>200	VL zenith <100.000
Gubavu *et al*. *JAC 2016*	F	21	47	38	2× ≥ 50 cp/ml	O	Yes	-	-	-	-	-
Oldenbüttel *et al*. *AVT 2016*	D	31	44.5	32	2× ≥ 20 cp/ml	O	Yes	Not to InSTI	-	≥ 6	-	no AIDS history
Rokx *et al*. *JAC 2016*	NL	5	63	0	2× ≥ 50 cp/ml	I	Yes	-	-	≥ 12	-	-
Rojas *et al*. *JAC 2016*	E	31	56	55	2× ≥ 37 cp/ml	O	Yes	-	-	-	-	-
Lecompte *et al*. *IAS 2017*	CH	8	44.5	28.5	1× ≥ 200cp/ml	I	No	No	≥ 24	-	-	-
Blanco *et al*. *JAC 2018*	E	31	47	10	2× ≥ 50 cp/ml or 1× > 1000 cp/ml	I	Yes	-	-	≥ 12	>200	-
**DTG-3TC**												
Borghetti *et al*. *JAC 2016*	I	36	53	19.4	2× ≥ 50 cp/ml	O	Yes	-	-		-	-
Maggiolo *et al*. *BMC ID 2017*	I	94	52	32.3	2× ≥ 50 cp/ml	O	Yes	Not to 3TC or InSTI	> 6	≥ 6	-	-
Joly *et al*. *CROI 2017*	F	104	45	14.4	2× ≥ 50 cp/ml	I	No	No	-	≥ 24	>200	no HIV encephalitis ^£^
Reynes *et al*. *HIV Glasgow* *2016*	F	27	59	25.9	2× ≥ 50 cp/ml	O	Yes	Not to InSTI	> 12	-	-	-
Blanco *et al*. *JAC 2018*	E	29	44	21		I	Yes	-	-	≥ 12	>200	-
Maggiolo *et al*. *EACS 2017*	I	203	52	24.6	-	O	Yes	No M184V	-	≥ 6	-	-
Taiwo *et al*. *CID 2017*	US	44	46	17	2× ≥ 50 cp/ml	I	No	No	≥ 12	≥ 12		
**DTG-RPV**												
Llibre *et al*. *Lancet 2018*	Multi- country	513	43	23	1× ≥ 50 cp/ml	I	No	-	> 6	≥ 12	-	-
Gantner *et al*. *HIV Med 2017*	F	116	55	44	2× ≥ 50 cp/ml or 1× ≥ 1,000 cp/ml	O	Yes	-	-	-	-	-
Bonijoly *et al*. *EACS 2017*	F	268	55	44	2× ≥ 50 cp/ml	O	Yes	-	-	≥ 6	-	On ART for ≥ 12 months
Revuelta *et al*. *Ann pharmacol* *2018*	E	32	49	37	2× ≥ 50 cp/ml	O	Yes	Not to InSTI or RPV	-	-	-	-
**DTG-ATV**												
Riva *et al*. *HIV Glasgow* *2016*	I	61	52.1	39	-	O	-	-	-	-	-	-
Castagna *et al*. *EACS 2017*	I	116	53	13	2× ≥ 50 cp/ml	O	-	-	-	≥ 12	-	-
**DTG-DRV**												
Navarro *et al*. *EACS 2017*	E	27	52	30	2× ≥ 50 cp/ml	O	Yes	Only to one ART class	-	≥ 6	-	-

Abbreviations: VF: virologic failure, ART: antiretroviral therapy, DTG: dolutegravir, 3TC: lamivudine, FTC: emtricitabine, RPV: rilpivirine, ATV: atazanavir, InSTI: integrase stand transfert inhibitor, F: France, NL: The Netherlands, D: Germany, E: Spain, CH: Switzerland, I: Italy, USA: United States of America, O: observational study; I: interventional study £:      no abnormal standard biological parameter

### Risk of bias

All RCTs were open-label non-inferiority trials ^8, 18, 35^, of which one was a single center trial ^18^ and three were multicenter trials ^8, 11, 35^. They reported adequate generation of random allocation sequences and allocation concealment. Three single-arm trials were included, of which two included less than 10 patients ^21, 24, 29^. All interventional studies adequately addressed incomplete outcome data: proportions of drop-outs were low and outcome data were missing for less than 20% of participants in all studies. Five of seven trials reported on virological outcomes at both time-points of interest for this study (24 and 48 weeks) ^8, 11, 24, 29, 35^. There was no evidence of selective reporting in any of the studies. In each of the 14 observational studies included in this review, the main demographic and clinical characteristics of the study populations were similar and patients were followed for 24 weeks in most studies. Among the observational studies, the majority did not report detailed inclusion and exclusion criteria. Five observational studies reported virological outcomes at both time-points ^12, 30– 33^. Amplification for drug resistance testing was successful for 19 of the 27 (70%) patients with VF. Finally, patient retention was over 90% in all 14 cohort studies.

### Virological and treatment failure

**Figure 2.**
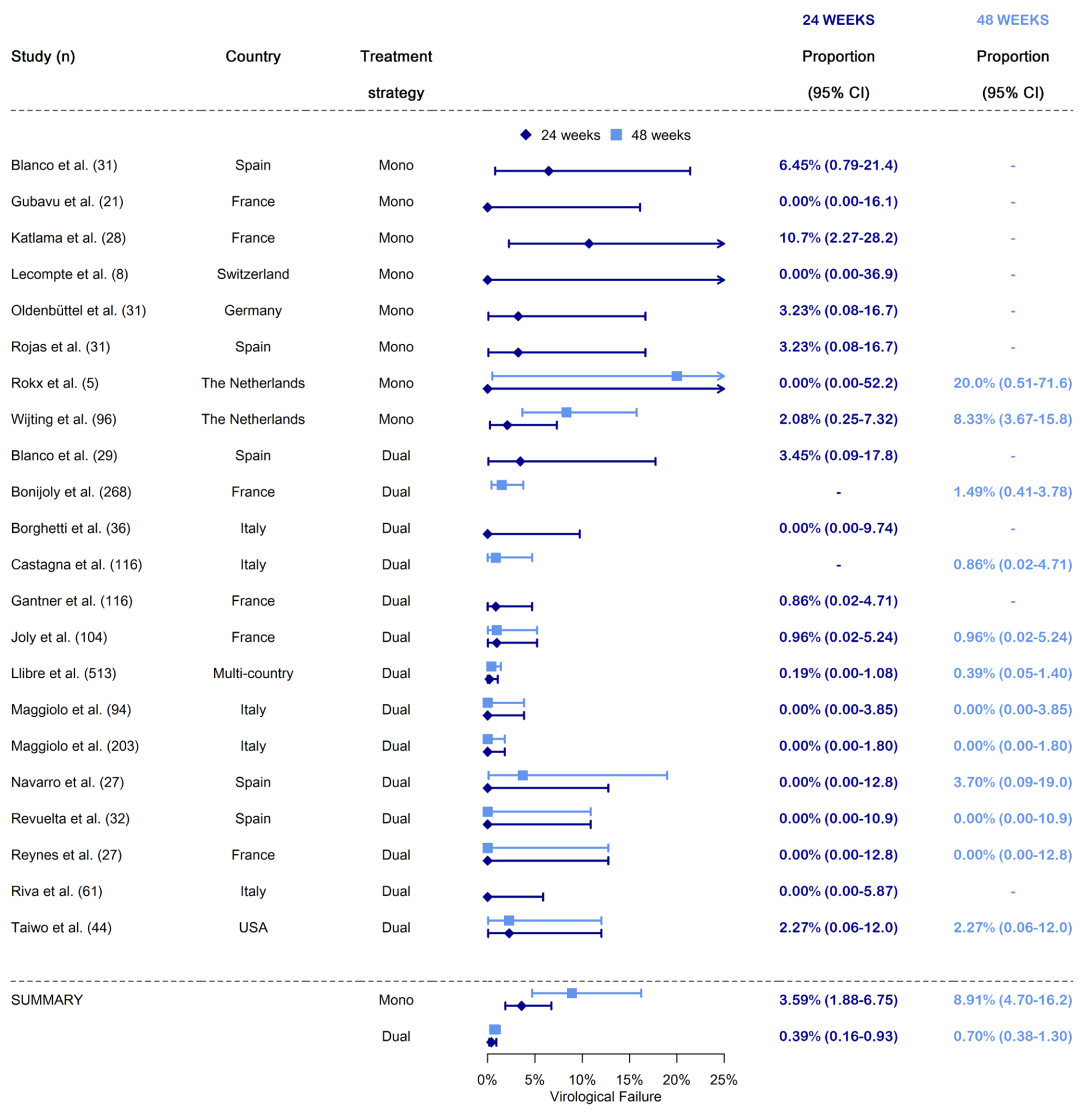
Meta-analysis of virological failure among patients on single or dual DTG-based simplification therapy.

The pooled estimate of the proportion of participants who experienced a VF on DTG-based monotherapy was 3.6% (95% CI 1.9-6.7) at 24 weeks and 8.9% (95% CI 4.7-16.2) at 48 weeks ( Figure 2). The high proportion of treatment failures among patients on monotherapy at 48 weeks was driven by the two studies from the Netherlands, which observed between 8 and 20% of VF ^8, 24^. Among patients on dual therapy, an estimated 0.4% (95% CI 0.2-0.9) experienced a VF at 24 weeks and 0.7% (95% CI 0.4-1.3) at 48 weeks. Independently of the combination used (DTG/3TC, DTG/RPV, DTG/ATV or DTG/DRV), 11 of 14 studies evaluating the effectiveness of dual therapy had less than 1% of patients developing VF. Compared to patients on monotherapy, those on dual therapy were less likely to experience VF by 24 weeks (odds ratio [OR] 0.10, 95% CI 0.03-0.32, p<0.001) and 48 weeks (OR 0.07, 95% CI 0.03-0.18, p<0.001). In analyses adjusted for study type (interventional or observational), age (median or mean) and sex (proportion of female participants), the OR for VF at 24 weeks and 48 weeks were very similar to the unadjusted estimates (aOR 0.10, 95% CI 0.03-0.30 for 24 weeks and aOR 0.06, 95% CI 0.01-0.30 for 48 weeks, respectively). The only variable that contributed to explaining the between-study heterogeneity in both the 24 and 48-week analyses was treatment strategy. When including this variable, the tau-squared were reduced from 1.17 (95% CI 0.33-2.19) to 0.00 (95% CI 0.00-1.11) in the 24 week analysis and from 1.37 (95% CI 0.54-2.15) to 0.00 (95% CI 0.00-1.00) in the 48 week analysis. The inclusion of other variables did not impact the estimates of tau-squared.

Treatment failure occurred in 5.2% (2.0–12.9) of patients at 24 weeks and 12.3% (4.5–29.4) at 48 weeks on DTG-monotherapy, whereas this outcome was observed in 2.8% (1.4–5.7) of patients at 24 weeks and 6.5% (4.3–9.6) at 48 weeks on DTG-based dual therapy. At 24 weeks, patients on dual therapy tended to be less likely to experience treatment failure compared to those on monotherapy (aOR 0.52, 95% CI 0.15-1.85). Due to multi-collinearity in the model, we were not able to report on multivariable analyses comparing treatment failure between mono and dual therapy at 48 weeks.

### Drug resistance

Acquired resistance mutations to InSTI developed in 9/251 (3.6%) participants on DTG-based monotherapy, which corresponded to 56% of the cases of VF ( Table 2). Three individuals developed the Q148R or Q148H mutation in combination with other resistance mutations, conferring high-level resistance to DTG ^20, 22^. Two of these three patients were previously exposed to InSTI and none had a history of previous VF. They all had a suppressed HIV viral load for several years before switching to DTG-monotherapy. No InSTI resistance mutations developed in patients on dual therapy. Of 962 patients on RPV/DTG, only one developed a major drug resistance mutation to non-nucleoside reverse transcriptase inhibitors (K101E). No resistance was observed in plasma among 237 individuals on DTG/3TC.

**Table 2.** Virological outcomes and drug resistance, by study.

Study	Follow-up (weeks)	N° patients	N° treatment failures (%)		N° virological failures (%)		N° amplified	N° patients with resistance	Resistance patterns (one line per patient) *
			24 weeks	48 weeks	24 weeks	48 weeks			
**DTG-Mono**									
Katlama *et al*.	24	28	4 (14.3)	-	3 (10.7)	-	3	**3**	**E138K,G140A, Q148R** **E92Q** **N155H**
Wijting *et al*.	48	96	-	11 (11.5)	2 (2.1)	8 (8.3)	6	**3**	**S230R** **R263K** **N155H**
Gubavu *et al*.	24	21	0	-	0	-	-	-	
Oldenbüttel *et al*.	24	31	2 (6.5)	-	1 (3.2)	-	1	**1**	**Q148H, G140S**
Rokx *et al*.	48	5	0	1 (20)	0	1 (20.0)	1	0	
Rojas *et al*.	24	31	1 (3.2)	-	1 (3.2)	-	1	0	118R **
Lecompte *et al*.	24	8	1 (12.5)	-	0	-	-	-	
Blanco *et al*.	24	31	2 (6.5)	-	2 (6.4)	-	2	**2**	**E138A, S147G, N155H,** **Q148R** **138K, 155H, 140S**
**DTG-3TC**									
Borghetti *et al*.	24	36	3 (8.3)	-	0	-	-	-	
Maggiolo *et al*.	48	94	0	3 (3.2)	0	0	-	-	
Joly *et al*.	48	104	1 (1.0)	3 (2.9)	1 (1.0)	1 (1.0)	0	-	
Reynes *et al*.	48	27	3 (11.1)	3 (11.1)	0	0	-	-	
Blanco *et al*.	24	29	1 (3.5)	-	1 (3.4)	-	1	0	K70E ***, K219E ***, G190R ^$^, M230I ^$^
Maggiolo *et al*.	48	203	0	12 (6.0)	0	0	-	-	
Taiwo *et al*.	48	44	1 (2.3)	3 (6.9)	1	1	1	0	
**DTG-RPV**									
Llibre *et al*.	48	513	-	27 (5.3)	1 (0.2)	2 (0.4)	2	1	K101K/E
Gantner *et al*.	24	116	11 (9.5)	-	1 (0.9)	-	0	-	
Bonijoly *et al*.	24	268	-	51 (19.0)	-	4 (1.5)	-	-	
Revuelta *et al*.	48	32	-	2 (6.5)	0	0	-	-	
**DTG-ATV**									
Riva *et al*.	24	61	3 (4.9)	-	0	-	-	-	
Castagna *et al*.	48	116	5 (4.3)	6 (5.2)	-	1 (0.9)	-	-	
**DTG-DRV**									
Navarro *et al*.	48	27	-	2 (7.4)	0	1 (3.7)	1	0	

*bold: InSTI resistance

**in 7% of integrated DNA in PBMC

*** in ≤1.5% of integrated DNA in PBMC

^$^ in integrated DNA in PBMC

Dolutegravir meta-analysis summary data This table shows summary measures, including the number of virological and treatment failures in each study. Click here for additional data file. Copyright: © 2019 Wandeler G et al. 2019 Data associated with the article are available under the terms of the Creative Commons Zero "No rights reserved" data waiver (CC0 1.0 Public domain dedication).

## Discussion

We performed a comprehensive systematic review of studies that reported on VF among patients switched to DTG-based maintenance therapy. Our meta-analysis shows that DTG-based dual therapy is successful in sustaining virological control in ART-experienced HIV-infected patients: only 12 of 1670 (0.7%) experienced a VF and none of them developed resistance mutations to DTG. On the contrary, 16 of 251 (6.4%) individuals switched to DTG monotherapy had a VF, of which more than one-half developed resistance to DTG. In comparison, recent trials evaluating InSTI-based triple maintenance combinations showed a similar virological failure rates ^36, 37^. Although the proportion of patients experiencing a confirmed viral rebound on DTG-monotherapy does not seem to be higher than in patients on PI-monotherapy, the risk of losing future treatment options is higher with DTG-monotherapy ^3^. Overall, our findings suggest that DTG-based monotherapy is not an appropriate simplification strategy and that further studies are urgently needed to confirm the long-term efficacy of DTG-based dual therapy.

DTG-based dual therapy is a promising simplification strategy, especially when combined with 3TC, as the likelihood of developing toxicity events and DDI on such regimens is very low. No drug resistance mutations to DTG developed among more than 1600 patients on dual therapy followed for 24 to 48 weeks and only one had a resistance mutation to another drug class. Although based on very few patients, the results seemed to be independent of previous virological failures. For instance, no virological failures were noted among patients on DTG/3TC despite the presence of a 184V mutation at the time of simplification in several studies. The impact of the latter mutation on viral fitness has been extensively described both *in vitro* ^38^ and *in vivo* ^39^, and could also potentially explain the improved treatment outcomes in these patients compared to those switched to DTG-monotherapy without any previous failures. Interestingly, similar observations were made for bPI-based regimens, for which efficacy was improved when 3TC was added, despite the presence of the 184V mutation ^39^.

We also report on estimates of treatment failure, which includes other reasons for treatment interruptions, such as toxicity or loss to follow-up. In our meta-analysis, the proportion of patients experiencing this combined outcome was more than twice as high among patients on monotherapy compared to those on DTG-based dual therapy. Although this outcome is important in evaluating the clinical efficacy of a novel ART strategy, our capacity to analyze this outcome in detail was limited by the missing information on the specific reasons for treatment interruptions in many studies and by the small number of events, especially at 48 weeks of therapy.

Of all simplification strategies evaluated to date, the DTG/3TC combination could be the one most readily accessible for patients in low- and middle-income countries: both DTG and 3TC are available and prequalified by stringent regulatory authorities in generic formulations. In order to be widely implemented, the efficacy of this dual combination should first be evaluated in large studies among different patient populations. The results from the studies included in our meta-analysis are mainly based on selected populations of HIV-infected individuals from European cohorts, and are not generalizable. Furthermore, long-term data are needed, as most treatment failures occurred after the first 24 weeks in several monotherapy studies. Recently, results from the only study which assessed 96-week outcomes with this regimen to date were reported: among 27 ART-experienced individuals with previous VF, DTG/3TC was 100% efficacious virologically ^12^. However, despite these encouraging results, data from larger studies are needed. In addition, more data on the activity of DTG-based simplified regimens in compartments other than blood are needed. Letendre *et al*. showed that DTG achieved therapeutic concentrations in the central nervous system (CNS), with a CNS penetration effectiveness score of four ^40^. In the MONODO study, all patients had an undetectable plasma HIV viral load at week 24 on DTG maintenance monotherapy, whereas only one had a detectable viral load in the cerebrospinal fluid ^21^. Moreover, levels of HIV-RNA in the genital tract on DTG monotherapy ^41^ and on DTG-3TC ^42^ were comparable to those on standard cART. However, these results were based on a very small sample of patients and data from individuals on simplified, DTG-based therapies are lacking.

As a wealth of data on the efficacy of DTG-maintenance strategies from small studies are being disseminated at a fast pace, this systematic review is the first analysis to provide comparative estimates of virological failure between DTG-based monotherapy and dual therapy. More than 1700 studies were screened, including abstracts from all important HIV conferences in the past years. As our meta-analysis included studies with diverse study designs and populations, it could be argued that the comparison of studies with such differences might be problematic. However, the estimates of VF were very similar across studies, especially in the DTG-based dual therapy arm. This finding highlights the potency of this combination, even in the presence of previous drug resistance mutations or multiple co-morbidities. Unfortunately, only studies including low numbers of patients reported outcomes from individuals on DTG-monotherapy, and data on dual therapy was dominated by one large study that assessed the efficacy of the DTG/RPV combination. As a consequence, the comparison of DTG-monotherapy vs. DTG/3TC, which would have been the most interesting one, was not possible. Furthermore, the lack of availability of individual data from the different studies precluded the analysis of risk factors of VF in the different simplification regimens. As most studies were observational, it is possible that the investigators mainly included patients with good adherence, which may have limited the generalizability of their findings. Finally, our results might have slightly under-estimated the proportion of patients with VF as individuals who were lost to follow-up might have experienced this outcome without them being accounted for. However, our treatment failure estimates showed that even when other reasons of treatment failure were considered, DTG-based dual therapy was superior to monotherapy.

In summary, DTG-based dual maintenance therapy seems to be a promising simplification strategy with high virological efficacy and low potential for DDI and toxicity. Such a treatment regimen could be an interesting alternative to classical triple-ART in selected patients. Furthermore, dual therapy might be a cost-effective global ART strategy ^43^. A number of large prospective studies evaluating the efficacy of DTG-based dual therapy are under way and will inform its potential implementation at a large scale. In addition to the studies using DTG-emtricitabine(FTC ^44^) and DTG-3TC ^45^ maintenance therapy, clinical trials are also assessing the efficacy of DTG/3TC in treatment-naïve patients ^46^. Furthermore, it will be critical to evaluate the efficacy of DTG-3TC or FTC dual therapy in specific sub-groups such as pregnant and breast-feeding women, adolescents, patients with previous failure to standard triple regimens and harboring the M184V resistance mutation, as well as in patients with HIV associated neurocognitive disorder and tuberculosis coinfection.

## Data availability

The data referenced by this article are under copyright with the following copyright statement: Copyright: © 2019 Wandeler G et al.

Data associated with the article are available under the terms of the Creative Commons Zero "No rights reserved" data waiver (CC0 1.0 Public domain dedication). 

F1000Research: Dataset 1. Dolutegravir meta-analysis summary data. 10.5256/f1000research.15995.d215724 ^47^

## Supplementary-material

**Supplementary File 1: Completed PRISMA checklist.**

**Supplementary File 2: Search strategy for Medline.**
