# Supplementary material for: Virologic failure and HIV drug resistance on simplified, dolutegravir-based maintenance therapy: Systematic review and meta-analysis
Source: F1000Res. 2019 Apr 3;7:1359. Originally published 2018 Aug 30. [Version 2] doi: 10.12688/f1000research.15995.2 (PMC6134332; doi:10.12688/f1000research.15995.2)
Supplement: Supplementary file 3 [file f1000research-7-19875-s0002.tgz › 3a7bf0cf-076d-4480-9ca3-fec7f12d27eb_Supplementary_File_2.docx]

**Supplementary File 2: Search strategy for Medline**

(("dolutegravir" [Supplementary Concept] ) OR "Heterocyclic Compounds, 3-Ring"[Mesh:NoExp] OR dolutegravir*[Title/Abstract]) AND (((((((HIV Infections[MeSH] OR HIV[MeSH] OR hiv[tiab] OR hiv-1*[tiab] OR hiv1[tiab] OR hiv infect*[tiab] OR human immunodeficiency virus[tiab] OR human immunedeficiency virus[tiab] OR human immuno-deficiency virus[tiab] OR human immune-deficiency virus[tiab] OR ((human immun*[tiab]) AND (deficiency virus[tiab])) OR acquired immunodeficiency syndrome[tiab] OR acquired immunedeficiency syndrome[tiab] OR acquired immuno-deficiency syndrome[tiab] OR acquired immune-deficiency syndrome[tiab] OR ((acquired immun*[tiab]) AND (deficiency syndrome[tiab])) OR "sexually transmitted diseases, Viral"[MeSH:noexp])))))))
