# Supplementary material for: Virologic failure and HIV drug resistance on simplified, dolutegravir-based maintenance therapy: Systematic review and meta-analysis
Source: F1000Res. 2019 Apr 3;7:1359. Originally published 2018 Aug 30. [Version 2] doi: 10.12688/f1000research.15995.2 (PMC6134332; doi:10.12688/f1000research.15995.2)
Supplement: Peer review history

# Peer review: Reviewer response for version 1

**DOI:** 10.5256/f1000research.17470.r37756

**Published:** 2018-09-10

**Authors:** Omar Sued

**Affiliations:** 1 Fundación Huésped, Buenos Aires, Argentina

The topic is extremely important, the conclusion is very relevant for informing the clinical practice and the timing is perfect. 

 Please see below some comments to improve this excellent systematic review

- Note that reference 9 is for naive patients, and not for switching. 
- REPRODUCIBILITY: Risk of bias checklist is not available. It could be good idea to include as supplementary material a sample of the checklist of assessment of bias. 
- The number of reviewed studies is 21, but the authors reported 8 for monotherapy and 14 for dual therapy without clarifying that some are focused on both strategies.
- Similarly, in figure 2, the name of the author is followed by the (n) of participants. This makes the reader think this is the reference. Please consider to add the reference number in this place, and an additional column for "n"
- Regarding the phrase "These three patients did not have a history of previous VF and had a suppressed HIV viral load for several years before switching to DTG-monotherapy". Please comment if patients experiencing INSTI resistance in monotherapy were previously exposed to INSTI. 
- Please review Table 2, because some INSTI mutations are not in bold, and the $ symbol is not explained in notes
- In discussion you mention "For instance, no virological failures were noted among patients on DTG/3TC despite the presence of a 184V mutation at the time of simplification in several studies." but you are not showing this result. Given the potential clinical importance of this issue, consider show how many participants had this mutation and show the outcomes.
- In the discussion, these phrases look similar:

1. First Paragraph: "Although the proportion of patients experiencing a confirmed viral rebound on DTG-monotherapy does not seem to be higher than in patients on PI-monotherapy, the risk of losing future treatment options is higher with DTG-monotherapy"
2. Second Paragraph "Although the proportion of patients experiencing a confirmed viral rebound on DTG-monotherapy does not seem to be higher than in patients on PI-monotherapy, their chances of losing future treatment options is higher than reported in most PI-monotherapy trials"

- Why to mention DTG-XTC if no study was presented with FTC?. I would suggest to stick to the presented data, therefore to discuss about 3TC.  
- The phrase "In addition to the studies on maintenance therapy ^39, 40^, clinical trials are also assessing the efficacy of DTG/XTC in treatment-naïve patients" should be updated based on the results of GEMINI1&2.
- It could be good to try to explain the higher rate of failure in those three trials in dual therapy (Blanco, and Navarro). Please check the failure rate in figure 2 for Taiwo (44 patients with failure 2.7%).

I have read this submission. I believe that I have an appropriate level of expertise to confirm that it is of an acceptable scientific standard.

# Response

**Published:** 2019-02-03

**Authors:** Gilles Wandeler

**Affiliations:** Bern University Hospital, Switzerland

Note that reference 9 is for naive patients, and not for switching.

*We agree with the comment. However, we prefer to keep this unchanged as we used the reference in a generic sentence.*

 REPRODUCIBILITY: Risk of bias checklist is not available. It could be good idea to include as supplementary material a sample of the checklist of assessment of bias.

*We used the Cochrane Collaboration list to assess risk of bias among the two RCT included in the meta-analysis. For observational studies, it was not appropriate to use the ROBINS-I tool, as we only considered data from the group of patients maintenance therapy. Thus, we assessed the population characteristics and missing outcome data for each study. This is explained in detail in the methods section of the published manuscript.*

 The number of reviewed studies is 21, but the authors reported 8 for monotherapy and 14 for dual therapy without clarifying that some are focused on both strategies.

*We explained this in the text (see data analysis section).*

 Similarly, in figure 2, the name of the author is followed by the (n) of participants. This makes the reader think this is the reference. Please consider to add the reference number in this place, and an additional column for "n"

*The reference is indicated differently in the text and we decided to keep the figure 2 unchanged.*

 Regarding the phrase (page 9) "These three patients did not have a history of previous VF and had a suppressed HIV viral load for several years before switching to DTG-monotherapy". Please comment if patients experiencing INSTI resistance in monotherapy were previously exposed to INSTI.

*We included this information in the new version of the paper (see drug resistance section).*

 Please review Table 2, because some INSTI mutations are not in bold, and the $ symbol is not explained in notes

*We adapted the table 2 in the new version of the paper.*

 In discussion you mention "For instance, no virological failures were noted among patients on DTG/3TC despite the presence of a 184V mutation at the time of simplification in several studies." but you are not showing this result. Given the potential clinical importance of this issue, consider show how many participants had this mutation and show the outcomes.

*As this information was missing from most studies, we described cases where reported but could not formally evaluate the association between specific mutations and treatment failure.*

 In the discussion, these phrases look similar:

 First Paragraph: "Although the proportion of patients experiencing a confirmed viral rebound on DTG-monotherapy does not seem to be higher than in patients on PI-monotherapy, the risk of losing future treatment options is higher with DTG-monotherapy"

 Second Paragraph "Although the proportion of patients experiencing a confirmed viral rebound on DTG-monotherapy does not seem to be higher than in patients on PI-monotherapy, their chances of losing future treatment options is higher than reported in most PI-monotherapy trials"

*Adapted in the text (doubled sentence cancelled from the 2nd paragraph of the discussion section).*

 Why to mention DTG-XTC if no study was presented with FTC?. I would suggest to stick to the presented data, therefore to discuss about 3TC.

*Adapted in the new version of the paper (see discussion section): XTC canceled and FTC used in reference to study using FTC.*

*The phrase "In addition to the studies on maintenance therapy 39, 40, clinical trials are also assessing the efficacy of DTG/XTC in treatment-naïve patients" should be updated based on the results of GEMINI1&2.*

*Adapted in the new version of the paper (see discussion section)*

 It could be good to try to explain the higher rate of failure in those three trials in dual therapy (Blanco, and Navarro).

*This is an important comment. However, available data within these studies did not allow us to make specific conclusions.*

*Blanco dual: 3.45% VF at W24.*

*Bonijoly: 1.49% VF at W48.*

*Navarro: 3.70% of VF at W48.*

*Taiwo: 2.27% VF at W 24 and W48*

*All others dual < 1% of VF both at 24 and 48 weeks.*

 Please check the failure rate in figure 2 for Taiwo (44 patients with failure 2.7%).

*We checked again data and did not find any error in the figure.*

*Only one of the 44 patients exposed to dual therapy had a rel VF (1/44 0 2.7%). with the FDA snapshot algorithm 2 other patients (one lost to FUP and one switching therapy because AE) were considered as failure by authors at W24.*

# Peer review: Reviewer response for version 1

**DOI:** 10.5256/f1000research.17470.r37757

**Published:** 2018-09-10

**Authors:** Laurent Hocqueloux

**Affiliations:** 1 Orleans Regional Hospital Centre (CHR Orléans), Orléans, France

Wandeler and colleagues provide here an excellent and comprehensive review on DTG-based maintenance therapy, even though this review will lack the latest communications on the topic (at IAS 2018). Nevertheless, the conclusions they make are in accordance with what is currently admitted by experts in the field and worldwide guidelines: DTG monotherapy lead to an unacceptable virologic failure (VF) rate (because of >50% of emerging mutations to the class) whereas dual therapy has an excellent efficacy and no VF with mutations to the class.

 I only have minor comments or questions:

 Results:

- (page 4) Can the authors provide any data on the impact of CD4 nadir on VF during monotherapy (as described by Wijting in the DOMONO trial)?
- (page 8) Authors should give more explicit conclusion on this paragraph “The only variable that contributed to explaining the between-study heterogeneity in both the 24 and 48-week analyses was treatment strategy. When including this variable, the tau-squared were reduced from 1.17 (95% CI 0.33-2.19) to 0.00 (95% CI 0.00-1.11) in the 24 week analysis and from 1.37 (95% CI 0.54-2.15) to 0.00 (95% CI 0.00-1.00) in the 48 week analysis. The inclusion of other variables did not impact the estimates of tau-squared.”

Discussion:

- (page 9) Please provide some references (at least one) for the impact of M184V on viral fitness (this one is of interest for DTG-based regimen: doi: 10.1097/QAD.0000000000001191)
- (page 9) I think authors should provide some data on VF (%, emerging mutations) during switch from a triple therapy to another one (in order to have an “historical comparator” for dual therapy)
- (page 10, “In addition, more data on the activity of DTG-based simplified regimens in compartments other than blood are needed.”) There are some references for mono- or dual-therapy in the genital tract (Hocqueloux et al. ^1^ and Gianella et al. ^2^)and CNS (Doco Lecompte et al. ^3^)
- (page 10) Authors should cite recent reports (all communicated at the IAS 2018 in Amsterdam) confirming their conclusions, even though they cannot include them in the analyses: two randomized-controlled clinical trials on DTG monotherapy (Braun et al. ^4^ and Hocqueloux et al. ^5^), the extended follow-up of the SWORD trials at week 100 (Aboud et al. ^6^) and results of the GEMINI trials (Cahn et al. ^7^ ).

References:

- (references 25 and 28) I think two references (Gantner and Bonijoly) are duplicates (as they are based on the analyze of the same database; Bonijoly et al. have included more patients / with longer duration of follow-up than Gantner).

I have read this submission. I believe that I have an appropriate level of expertise to confirm that it is of an acceptable scientific standard.

## References

[1] HocquelouxCGubavuSDe DieuleveultBBuretJMilleCRouziouxCPrazuckTAvettand-FenoelV: HIV-1-infected patients under successful less-drug regimens have similar genital shedding and residual viremia than those under triple therapy. *IAS Conference on HIV Science*.2017; Reference source

[2] GianellaSWilkinTBerzinsBSaxP.EMoserCMarconiVFichtenbaumC.JBensonC.AVargasMOliveiraM.FTaiwoB.O: Genital HIV-1 shedding with Dolutegravir (DTG) plus Lamivudine (3TC) dual therapy.2018; Reference source 10.1097/QAI.0000000000001863PMC623195230383591

[3] Doco LecompteTSculierDyerlySDecosterdLNawejODimitrovaYCalmyA: MONODO: peripheral blood and cerebrospinal fluid viremia of 24-weeks MONOtherapy of DOlutegravir in HIV-1 virologically suppressed patients.2017; Reference source

[4] BraunD.LTurkTHampleBGrubeCSchrieberPGreinerMSteffensDTschumiFBayard deTorrentéCDepmeierCMetznerKBertischBBöniJKouyosRGünthardR: Simplification to dolutegravir monotherapy is non-inferior compared to continuation of combination antiretroviral therapy in patients who initiated combination antiretroviral therapy during primary HIV infection: A randomized, controlled, non-inferiority trial.2018; Reference source

[5] HocquelouxLAllavenaCPrazuckTBernardLSunderSEsnaultJ.LReyDLe MoalGRoncato-SaberanMAndréMBillaudEAvettand-FénoëlVValéyARaffiFParientiJ.J: Dolutegravir monotherapy versus dolutegravir/abacavir/lamivudine for HIV-1-infected virologically suppressed patients: Results from the randomized non-inferiority MONCAY trial.2018; Reference source

[6] Durable suppression 2 years after switch to DTG RPV 2-drug regimen: SWORD 1.

[7] CahnPSierra MaderoJArribasJAntinoriAOrtizRClarkeAHungC.CRochstrohRGiradP.mManCSieversJCurrieAUnderwoodMTenorioAPappaKWynneBGartlandMAboudMSmithK: Non-inferior efficacy of dolutegravir (DTG) plus lamivudine (3TC) versus DTG plus tenofovir/emtricitabine (TDF/FTC) fixed-dose combination in antiretroviral treatment-naïve adults with HIV-1 infection - 48-week results from the GEMINI studies.2018; Reference source

# Response

**Published:** 2019-02-03

**Authors:** Gilles Wandeler

**Affiliations:** Bern University Hospital, Switzerland

(page 4) Can the authors provide any data on the impact of CD4 nadir on VF during monotherapy (as described by Wijting in the DOMONO trial)?

*Unfortunately, we do not have these data from most of the studies.*

 (page 8) Authors should give more explicit conclusion on this paragraph “The only variable that contributed to explaining the between-study heterogeneity in both the 24 and 48-week analyses was treatment strategy. When including this variable, the tau-squared were reduced from 1.17 (95% CI 0.33-2.19) to 0.00 (95% CI 0.00-1.11) in the 24 week analysis and from 1.37 (95% CI 0.54-2.15) to 0.00 (95% CI 0.00-1.00) in the 48 week analysis. The inclusion of other variables did not impact the estimates of tau-squared.”

*The first sentence says explicitly that only the treatment strategy was found to contribute to heterogeneity, and we are not sure how to make the conclusion more explicit.*

*The second sentence quantifies the statement.*

 (page 9) Please provide some references (at least one) for the impact of M184V on viral fitness (this one is of interest for DTG-based regimen: doi: 10.1097/QAD.0000000000001191)

*We included references in the new version of the paper (see 2 ^nd^ paragraph in the discussion section).* 

 (page 9) I think authors should provide some data on VF (%, emerging mutations) during switch from a triple therapy to another one (in order to have an “historical comparator” for dual therapy)

*We added a comment in the 1st paragraph of the discussion section.*

*« We performed a comprehensive systematic review of studies that reported on VF among patients switched to DTG-based maintenance therapy. Our meta-analysis shows that DTG-based dual therapy is successful in sustaining virological control in ART-experienced HIV-infected patients: only 12 of 1670 (0.7%) experienced a VF and none of them developed resistance mutations to DTG. For comparison, recent trials testing InSTI-based triple maintenance combinations showed a similar virological failure rate (ref ci dessous 1-4). On the contrary, 16 of 251 (6.4%) individuals switched to DTG monotherapy had a VF, of which more than one-half developed resistance to DTG. »*

 (page 10) “In addition, more data on the activity of DTG-based simplified regimens in compartments other than blood are needed.” There are some references for mono- or dual-therapy in the genital tract (Hocqueloux et al. 1 and Gianella et al. 2) and CNS (Doco Lecompte et al. 3)

*We modified the text in the new version of the paper (see 4 ^th^ paragraph of the discussion section).*

 « In addition, more data on the activity of DTG-based simplified regimens in compartments other than blood are needed. Letendre et al. Showed that DTG achieved therapeutic concentrations in the central nervous system (CNS), with a CNS penetration effectiveness

 score of four. In the MONODO study, all patients had an undetectable plasma HIV viral load at week 24 on DTG maintenance monotherapy, whereas only one had a detectable viral load in the cerebrospinal fluid ( *ref 21 by Sculier et al*). Moreover, levels of HIV-1 RNA in the genital tract on DTG-monotherapy *(nouvelle ref 1 )* and under DTG-3TC *(nouvelle ref 2) *were comparable to those under standard cART. However, these results were based on a very small sample of patients and data from individuals on simplified, DTG-based therapies are lacking. »

*(*page 10) Authors should cite recent reports (all communicated at the IAS 2018 in Amsterdam) confirming their conclusions, even though they cannot include them in the analyses: two randomized-controlled clinical trials on DTG monotherapy (Braun et al. 4 and Hocqueloux et al. 5), the extended follow-up of the SWORD trials at week 100 (Aboud et al. 6) and results of the GEMINI trials (Cahn et al. 7).

*In accordance  with the pre-specified analysis plan described in our study protocol, we decided not to include these results as they had not been presented at the time of the last version of our analyses.*

 References: (references 25 and 28) I think two references (Gantner and Bonijoly) are duplicates (as they are based on the analyze of the same database; Bonijoly et al. have included more patients / with longer duration of follow-up than Gantner).

*We agree with the reviewer. However, as one study reported 24 weeks and the other 48 weeks outcomes, we decided to keep both estimates. Furthermore, we did not combine them as the study populations were not exactly the same.*

# Peer review: Reviewer response for version 1

**DOI:** 10.5256/f1000research.17470.r37755

**Published:** 2018-09-03

**Authors:** Esteban Martinez

**Affiliations:** 1 Hospital Clinic of Barcelona, University of Barcelona, Barcelona, Spain

This paper compared the efficacy of dolutegravir-based monotherapy vs dual therapy using the methodology of systematic review and meta-analysis. The topic is of great interest as many different studies with these simplication strategies have been done. Although dolutegravir monotherapy is not recommended at present due to the risk of virological failure with development of resistance mutations and dolutegravir dual therapy seems a promising strategy with recent evidence from large clinical trials, this systematic review and meta-analysis is timely because there are almost no direct comparisons between dolutegravir-based monotherapy vs dual therapy. The design and the methods (including PRISMA reporting) are adequate, as they are the interpretation of results. It is interesting that not only monotherapy was inferior to dual therapy but the difference resulted highly increased from 24 weeks to 48 weeks of follow-up, thus indicating that the risk of failure with the monotherapy strategy may greatly increase after the initial 24 weeks of follow-up. This is remarkable as many exploratory studies had 24-week results only.

I have read this submission. I believe that I have an appropriate level of expertise to confirm that it is of an acceptable scientific standard.

# Response

**Published:** 2019-02-03

**Authors:** Gilles Wandeler

**Affiliations:** Bern University Hospital, Switzerland

No comments needing to be addressed
